# Pulmonary Histoplasmosis in a Referral Hospital in Mexico City

**DOI:** 10.1155/2022/2121714

**Published:** 2022-06-24

**Authors:** Alejandro Hernández Solís, Javier Araiza Santibáñez, Jazmín Guadalupe Tejeda Olán, Andrea Quintana Martínez, Alejandro Hernández de la Torriente, Rocio de la Torriente Mata

**Affiliations:** ^1^Servicio de Neumología y Cirugía de Tórax, Hospital General de México “Dr. Eduardo Liceaga”, Ciudad de México, Mexico; ^2^Laboratorio de Micología, Hospital General de México “Dr. Eduardo Liceaga”, Ciudad de México, Mexico; ^3^Facultad de Estudios Superiores Cuautitlán Izcalli, Medicina Veterinaria y Zootecnia, Universidad Nacional Autónoma de México, Ciudad de México, Mexico; ^4^IMSS, Coordinación de Educación en Salud, División de Programas Educativos, Instituto Mexicano del Seguro Social, Ciudad de México, Mexico

## Abstract

Pulmonary histoplasmosis is caused by inhaling *Histoplasma capsulatum*. Less than 1% develops the disease. Risk factors in immunocompetent individuals are environmental exposures in endemic areas. The objective of this study is to determine the frequency, clinical, and microbiological characteristics in immunocompetent patients. A retrospective case series study of patients diagnosed with pulmonary histoplasmosis was performed in a respiratory care unit in Mexico City from 2000 to 2020. Each patient had bronchial lavage, and three patients underwent thoracoscopy for the lung tissue sample taken for the culture in Sabouraud Dextrose Agar. Twelve patients were identified, 8 males and 4 females; the predominant symptoms were fever (83%), dyspnea (75%), chest pain (66%), hemoptysis (41%), and weight loss (33%). The computed tomography of the chest showed the following findings: patchy consolidation 12 (100%), hilar adenopathy 6 (50%), pleural effusion 6 (50%), caverns 3 (25%), and solitary pulmonary nodule in one patient (8%). *Histoplasma capsulatum* was found in the culture of all twelve patients. The signs and symptoms of the disease are mediated by the immune status of the host. The clinical picture is often confused with systemic diseases. It is important to have a high degree of clinical suspicion to make a timely diagnosis.

## 1. Introduction

Pulmonary histoplasmosis (PH) is caused by *Histoplasma capsulatum*. It exists in the environment in the form of hyphae, microconidia, macroconidia, and in tissues such as yeast. Microconidia (spores) are the infectious form, and their inhalation is the main route of infection. Less than 1% of those exposed to the spores develop this disease; the development of symptoms depends on the level of exposure and the immune status of the exposed person, considering an endemic disease [1]. The incidence ranges from 0.1 to 1 case per 100,000 inhabitants per year and more than 100 cases per 100,000 in high-risk groups and during outbreaks [2]. Infections are associated with contamination of the soil by bat droppings. The clinical presentations in immunocompetent individuals at the pulmonary level are benign, mainly pulmonary nodules. It is common in the US and tropical areas of Mexico, Central America, and South America [3]. Unfortunately, in Mexico, invasive mycoses are not considered mandatory notifiable diseases, making it difficult to know the epidemiology of this disease [4]. The objective of the study is to determine the frequency and clinical characteristics of immunocompetent patients with PH in a respiratory care unit in Mexico City.

## 2. Materials and Methods

A retrospective, observational case series study was performed in a public assistance hospital. Clinical records of patients with a PH diagnosis, hospitalized for symptoms characterized by cough, dyspnea, chest pain, hemoptysis, fever, and presenting a chest X-ray scan with evidence of pulmonary consolidation, who were admitted to a respiratory care unit in Mexico City were studied from 2000 to 2020.

The histoplasmin test and bronchoalveolar lavage were performed on each patient with a diagnosis of PH using a flexible bronchoscope, obtaining 10 ml; 3 patients underwent thoracoscopic surgery obtaining biopsies of lung tissue. The biological material was processed in the Mycology Laboratory of the Hospital General de Mexico “Dr. Eduardo Liceaga”.

The sputum samples and surgical specimens were processed by direct examination with 10% of potassium hydroxide (KOH) and cultured in the Sabouraud dextrose agar medium incubated for 7 days at 28°C. They were observed macroscopically and microscopically to corroborate the presence of yeast, and special stains such as periodic acid Schiff, colloidal iron, and cotton blue lactophenol were performed.

## 3. Results

Twelve patients with PH diagnosis were identified, 8 males (66%) and 4 females (33%); the average age was 47+/−8, negative human immunodeficiency virus(HIV), without risk factors for immunosuppression. The symptoms were fever (83%), dyspnea (75%), chest pain (66%), hemoptysis (41%), and weight loss (33%). The cultures of bronchoalveolar lavage were positive in all patients (100%), as well as in the lung tissue biopsies of 3 patients applying thoracoscopy. The histoplasmin test was positive in 8 patients within 48 to 72 hours and erythema and induration lasted an average of 7 days.

They identified 5 (41%) patients with neutropenia; three of them were classified as moderate neutropenia <1000 cells/mm^3^ and two with severe neutropenia <500 cells/mm^3^, and neutropenia was caused by histoplasmosis infection.

**Figure 1:**
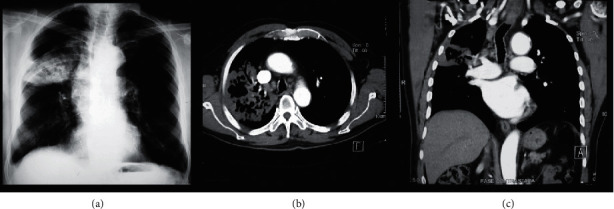
Necrotizing pneumonia due to *Histoplasma capsulatum*. (a) Chest X-ray (PA), with the presence of heterogeneous consolidation with radio lucid areas inside. (b, c) Chest CT showing lobar consolidation, cavitation in the right upper lobe, and parahilar lymphadenopathy.

The findings identified in the chest computed tomography were patchy consolidation 12 (100%), hilar adenopathy 6 (50%), exudative or hemorrhagic pleural effusion 6 (50%), caverns 3 (25%), and solitary pulmonary nodule in one patient (8%) (Figure 1 and Table 1).

**Figure 2:**
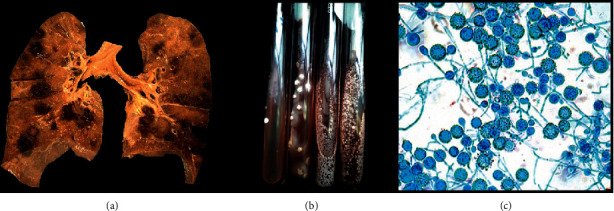
(a) Multiple nodular, hemorrhagic, and cavitated lesions scattered throughout the lung parenchyma. (b) Sabouraud's dextrose agar medium observing white, downy-looking colonies. (c) Direct examination with lactophenol cotton blue, in which thin, septate, hyaline hyphae with round, spiculated macroconidia were observed in a 40X image.

The lung tissue and bronchial lavage samples were cultured in Sabouraud dextrose agar at 28°C for 7 days. Limited hairy-looking white colonies were obtained without diffusible pigment to the medium. Cotton blue staining was performed with lactophenol observing thin, septate, hyaline hyphae with round, spiculated macroconidia in a 40X image. The identified species was *Histoplasma capsulatum* in 100% of the cultures; three of the patients (25%) died from hemoptysis (Figure 2).

## 4. Discussion

In Mexico, *Histoplasma capsulatum* has been isolated from the intestine, lung, liver, and spleen of bats, belonging to six different species in the central area of the country, in caves and mines, with the predominance of the genus *Natalusstramineus* (funnel ear) belonging to the *Natalidae* family from northern Mexico to Paraguay [5].

It is an opportunistic disease; after inhalation of hyphae and conidia, these are introduced into the bronchioles and alveoli, infecting the respiratory tract. Macrophages recognize and engulf the conidia that will become yeast. In the first weeks, yeasts multiply in alveolar macrophages and spread throughout the reticulum endothelial system, generating an immune response mediated by T cells. If the cellular response is not sufficient, the agent proliferates, causing a progressive and lethal spread [6].

Traveling to endemic areas or being in contact with guano birds or bats, use of steroids, diabetes mellitus, and HIV/AIDS with CD4 < 150 cells/mm^3^are risk factors especially in immunocompetent patients [7]. It generally occurs in immunosuppressed patients and is uncommon in those who do not have risk factors. In our study, 8 patients lived in endemic areas of the country, and 4 practiced speleology [8].

The cases of histoplasmosis in immunocompetent patients are not diagnosed as they are asymptomatic or present mild symptoms, self-limiting in 95%. The infection can occur by novel or by reactivation even years after infection. In our series, 75% presented mild symptoms, predominantly fever and dyspnea with medium effort [9].

In immunosuppressed individuals, it spreads to the bone tissue, spleen, and liver, producing progressive disseminated histoplasmosis, while in immunocompetent patients it is uncommon. More than 80% are located in the lungs, so it should be suspected in any traveler with fever, systemic symptoms, or community-acquired pneumonia [10].

The disease can be confused with lymphoma, lung metastases, or *tuberculosis* delaying treatment. If these symptoms become chronic, there is a loss of lung function with a mortality of 30%. In our series, no case was suspected to have the onset of PH admitted with a diagnosis of bacterial pneumonia and primary lung cancer; for differential diagnosis, all patients underwent GeneXpert, and all in all, the result was negative [11].

In Brazil, a study was performed to estimate the frequency of pulmonary mycoses in 213 smear-negative *tuberculosis* patients, and in 7% of the cases (15/213), pulmonary mycoses were diagnosed, comprising ten aspergillosis cases, three cases of paracoccidioidomycosis, one case of histoplasmosis, and one of cryptococcosis [12].

Symptoms in the acute pulmonary form appear in 1-2 weeks and present as cough, dyspnea, malaise, fever and chills, chest pain, and lymph nodes, persisting for 2 weeks causing misdiagnosis as bacterial pneumonia or viral respiratory disease. In the subacute form, there is a slow and progressive development for several weeks. The chronic form implies an evolution for months and the progressive histoplasmosis is disseminated and is characterized to occur in immunosuppressed individuals. However, a large inoculum can trigger severe disease in 29% of immunocompetent individuals, as presented in our patients who developed lymphadenopathy 7/12, hepatomegaly 5/12, and splenomegaly 2/12.

There are alterations in laboratory tests; being nonspecific findings, tests usually show anemia, leukopenia, thrombocytopenia, and elevated liver enzymes. In our series, neutropenia predominated in 41%, two patients with severe neutropenia <500 cells/mm^3^, and 5 patients with abnormal liver function tests [13].

In the chest radiography, we can find focal or patchy opacities, bilateral diffuse opacities, diffuse micronodular or air-space opacities, hilar or mediastinal lymphadenopathy, consolidation, calcifications, fibrosis, and cavities most commonly in the upper lobes. The first imaging study in our series was a chest radiograph scan, and predominant images were bilateral diffuse opacities and hilar lymphadenopathy which suggested infection caused by opportunistic organisms. The images that predominated in the chest CT scan in acute pulmonary histoplasmosis are patchy consolidation involving one or several lobes, concomitant hilar and mediastinal lymphadenopathy being common, solitary pulmonary nodules or multiple nodules with a nonspecific appearance, and occasionally demonstrate cavitation or ground glass halo; pericardial and pleural effusions are rare, and they often occur in younger patients, they are self-limited in almost all cases, and they are thought to have a hypersensitive reaction to *H. capsulatum* antigens [14]. In our series, pleural effusion was present in 50% of the patients, 25% of the patients were self-limited. Chronic cavitary pulmonary disease is an uncommon manifestation, and it is most exclusively seen in older white men with emphysema being the main risk factor [15]. The typical early imaging manifestation of chronic histoplasmosis is a segmental, wedge-shaped area of peripheral consolidation that has a moth-eaten appearance from the scattered foci of the emphysematous lung. Similar to pulmonary *tuberculosis* and chronic pulmonary aspergillosis, the cavitary lesion usually involves the apical and posterior segments of the upper lobes, especially the right upper lobe, and pleural thickening adjacent to apical cavitary lesions is common. In our series, 2 patients were presented with chronic histoplasmosis with a cavitary lesion, both in the right upper lobe [14] (Table 2).

The diagnosis includes the histopathological study, culture, antigen, and antibody detection. In acute pulmonary histoplasmosis, rapid diagnosis is critical and often possible by detection of antigen, but this test may be falsely negative in 17% of such cases; negative results do not exclude histoplasmosis. Repeated testing is advised for patients with progressive illness if the initial test results are negative. In disseminated histoplasmosis, the sensitivity of antigen detection is higher in immunocompromised patients than in immunocompetent patients and in patients with more severe illnesses [16].

In a study performed by Sarah M. Richer et al., the MiraVistaDiagnostics (MVista) histoplasma antibody enzyme immunoassay (EIA) showed high sensitivity (88.8%) and it offers increased sensitivity over current antibody tests while also allowing separate detection of IgG and IgM antibodies and complementing antigen detection. Combining antigen and EIA antibody testing provides an optimal method for diagnosis of acute pulmonary histoplasmosis [17].

In addition, patients with indeterminate pulmonary nodules in endemic areas can be benefited from the use of histoplasma IgG and IgM antibodies measured using EIA testing, and these antibodies potentially decrease unnecessary invasive biopsies for the diagnosis of chronic histoplasmosis [18].

In our patients, bronchoscopy turned out to be an ideal diagnostic method for obtaining biological material, as well as lung tissue obtained by thoracoscopy, having isolation in culture in all cases, being the gold standard for diagnosis. The histoplasmin test was positive in 66% of the cases [19]. Antibody and antigen tests were not performed.

The most frequent complications are mediastinal adenitis, pulmonary nodules (histoplasmoma), mediastinal granuloma, fibrosis, and recurrent hemoptysis, and the predominant sequela being fibrosis and caverns in 50% of the patients.

It has been described as a relationship between smokers with underlying chronic obstructive pulmonary disease (COPD) and chronic cavitary histoplasmosis, but it is not consistent; in a current series, 46 patients were included with a diagnosis of histoplasmosis with symptoms for>6 weeks. Only 39% had cavitary disease of which 72% were male and 89% were smokers with a high average number of 40 pack years. However, only 20% had a clinical diagnosis of COPD [20]. In our study, 3 patients were smokers and no one has a history of COPD.

In the presence of dyspnea, spirometry was performed at one year of follow-up, presenting in all cases a restrictive pattern with vital capacity (CV) < 60% [2, 3].

Treatment depends on the severity of the disease and lasts until symptoms resolve. Acute cases of primary infection should not be treated unless they are symptomatic and have extrapulmonary lesions; therefore, they are subclinical and self-limited. In severe forms, amphotericin B is indicated, which is subsequently continued with itraconazole. In acute respiratory distress syndrome, steroids are beneficial. In a chronic form, itraconazole is indicated. Relapses occur in 20%, which recommends follow-up for 2 years. In our study, 75% responded to treatment without presenting relapses and three died on average within 18 days after diagnosis due to intractable hemoptysis not responding to bronchial embolization [21] (Table 3).

## 5. Conclusion

During the study, less than 5% of hospital admissions to the respiratory care unit represented diagnosis of mycosis, and it should be considered to include fungal diseases such as pulmonary histoplasmosis as a differential diagnosis if a patient presents fever of unknown origin, even in immunocompetent patients, especially with a history of travelling to endemic areas. PH causes significant morbidity, and its prevalence remains unknown.

## Figures and Tables

**Table 1:** Patients with acute histoplasmosis.

Age/gender	Risk factors	Days onset	Symptoms	Imaging CT	Diagnosis	Treatment	Follow-up
42male	Practice speleology, endemic area	18	Fever, dyspnea, chest pain	Consolidation, pleural effusion, HA	Bronchoalveolar lavage, histoplasmin +	Itraconazole for 12 weeks	1 year
38female	Endemic area	10	Fever, chest pain	Consolidation, pleural effusion	Bronchoalveolar lavage, histoplasmin +	Itraconazole for 6 weeks	6 months
56male	Smoking, endemic area	6	Fever, dyspnea, hemoptysis, weight loss	Consolidation, solitary pulmonary nodule, HA	Bronchoalveolar lavage and thoracoscopy biopsy	AmB, methylprednisolone	Died
51female	Wood smoke	5	Fever, dyspnea, chest pain	Consolidation, pleural effusion	Bronchoalveolar lavage	Itraconazole for 12 weeks	1 year
55male	Smoking	8	Dyspnea, hemoptysis, chest pain	Consolidation, pleural effusion	Histoplasmin +, bronchoalveolar lavage	AmB for 2 weeks followed by itraconazole for 10 weeks methylprednisolone	1 year
49female	Endemic area	11	Fever, hemoptysis	Consolidation, hilar adenopathy	Histoplasmin +, bronchoalveolar lavage	Itraconazole for 6 weeks	6 months
40male	Practice speleology, endemic area	7	Fever, dyspnea, chest pain	Consolidation, pleural effusion	Histoplasmin +, bronchoalveolar lavage	Itraconazole for 12 weeks	1 year
50female	None	13	Fever, dyspnea, chest pain	Consolidation, pleural effusion, HA	Histoplasmin +, bronchoalveolar lavage	Itraconazole for 6 weeks	6 months
47male	Practice speleology, endemic area	4	Fever, chest pain, weight loss	Consolidation, pleural effusion	Histoplasmin +, bronchoalveolar lavage	Itraconazole for 12 weeks	1 year
41male	Practice speleology, endemic area	29	Dyspnea, hemoptysis, weight loss	Consolidation, cavern, HA	Bronchoalveolar lavage and thoracoscopy biopsy	AmB	Died

HA : Hilar adenopathy; AmB: amphotericin B.

**Table 2:** Patients with chronic histoplasmosis.

Patients	Risk factors	Days onset	Symptoms	Imaging CT	Diagnosis	Treatment	Follow-up
55male	Smoking	6 months	Fever, dyspnea, weight loss	Consolidation, cavern	Thoracoscopy biopsy and bronchoalveolar lavage	Itraconazole for 12 months	1 year
57male	Endemic area	4 months	Fever, dyspnea, hemoptysis, chest pain	Consolidation, cavern, HA	Histoplasmin +, bronchoalveolar lavage	Itraconazole	Died

HA : Hilar adenopathy.

**Table 3:** Characteristics presented in immunocompetent patients with pulmonary histoplasmosis.

Characteristics	*n*	(%)
Patients		
Male	8	(66%)
Female	4	(33%)

Symptoms		
Fever	10	(83%)
Dyspnea	9	(75%)
Chest pain	8	(66%)
Hemoptysis	5	(41%)
Weight loss	4	(33%)

Findings		
Lymphadenopathy	7	(58%)
Hepatomegaly	5	(41%)
Splenomegaly	2	(16%)

Laboratory findings		
Neutropenia	5	(41%)
Moderate	3	(25%)
Severe	2	(16%)
Abnormal liver function tests	5	(41%)

Chest imaging		
Consolidation	12	(100%)
Hilar adenopathy	6	(50%)
Pleural effusion	6	(50%)
Caverns	3	(25%)
Solitary pulmonary nodule	1	(8%)

Mortality		
Dead	3	(25%)

## Data Availability

The data used to support the findings of this study are available from the corresponding author upon request.
